# Quantification of tumor induced motor cortical plasticity using navigated transcranial magnetic stimulation in patients with adult-type diffuse gliomas

**DOI:** 10.3389/fnins.2023.1143072

**Published:** 2023-03-15

**Authors:** Cesar Cimonari de Almeida, Iuri Santana Neville, Cintya Yukie Hayashi, Alexandra Gomes dos Santos, André Russowsky Brunoni, Manoel Jacobsen Teixeira, Wellingson Silva Paiva

**Affiliations:** ^1^Division of Neurosurgery, Department of Neurology, University of São Paulo Medical School, São Paulo, Brazil; ^2^Service of Interdisciplinary Neuromodulation, Instituto de Psiquiatria do Hospital das Clínicas da Faculdade de Medicina da Universidade de São Paulo, São Paulo, Brazil; ^3^Instituto do Câncer do Estado de São Paulo, Hospital das Clínicas da Faculdade de Medicina da Universidade de São Paulo, São Paulo, Brazil

**Keywords:** transcranial magnetic stimulation, glioma, neurosurgery, neuronavigation guided, brain neoplasms, neuronal plasticity (MeSH), cerebral cortex

## Abstract

**Introduction:**

The evaluation of brain plasticity can provide relevant information for the surgical planning of patients with brain tumors, especially when it comes to intrinsic lesions such as gliomas. Neuronavigated transcranial magnetic stimulation (nTMS) is a non-invasive tool capable of providing information about the functional map of the cerebral cortex. Although nTMS presents a good correlation with invasive intraoperative techniques, the measurement of plasticity still needs standardization. The present study evaluated objective and graphic parameters in the quantification and qualification of brain plasticity in adult patients with gliomas in the vicinity of the motor area.

**Methods:**

This is a prospective observational study that included 35 patients with a radiological diagnosis of glioma who underwent standard surgical treatment. nTMS was performed with a focus on the motor area of the upper limbs in both the affected and healthy cerebral hemispheres in all patients to obtain data on motor thresholds (MT) and graphical evaluation by three-dimensional reconstruction and mathematical analysis of parameters related to the location and displacement of the motor centers of gravity (ΔL), dispersion (SDpc) and variability (VCpc) of the points where there was a positive motor response. Data were compared according to the ratios between the hemispheres of each patient and stratified according to the final pathology diagnosis.

**Results:**

The final sample consisted of 14 patients with a radiological diagnosis of low-grade glioma (LGG), of which 11 were consistent with the final pathology diagnosis. The normalized interhemispheric ratios of ΔL, SDpc, VCpc, and MT were significantly relevant for the quantification of plasticity (*p* < 0.001). The graphic reconstruction allows the qualitative evaluation of this plasticity.

**Conclusion:**

The nTMS was able to quantitatively and qualitatively demonstrate the occurrence of brain plasticity induced by an intrinsic brain tumor. The graphic evaluation allowed the observation of useful characteristics for the operative planning, while the mathematical analysis made it possible to quantify the magnitude of the plasticity.

## Introduction

The relationship between morphological and functional neuroanatomy has been known for millennia (Duque-Parra, 2001) giving birth to maps of brain function (Sabbatini, 2004). During this time the observations of dynamic changes in this morpho-functional correlation has been described through concepts of neuroplasticity (Rosenzweig, 1996; Costandi, 2016). Several types of brain injuries can induce plasticity, such as vascular accidents, trauma, malformations, and neoplasms. While acute events offer a good perspective on areas directly related to primary functions, congenital malformations and progressive lesions allow an assessment of the intrinsic capacity of the neurodevelopmental brain to reorganize functions based on the absence or anomalies of anatomical structures (Herbet et al., 2016; Bourdillon et al., 2017; Mateos-Aparicio and Rodríguez-Moreno, 2019).

The evaluation of brain plasticity can provide useful information for treatment planning, as well as adjust prognostic expectations. In this sense, intracranial tumors, especially intrinsic lesions, due to their progressive nature, offer both a good field of study and a candidate to benefit from this information on neuroplasticity (Duffau, 2005; Desmurget et al., 2007; Fisicaro et al., 2016; Takakura et al., 2017).

The use of non-invasive methods for cortical mapping has captured increasing attention during the last decades with the navigated transcranial magnetic stimulation (nTMS) being one of the most used techniques, specially for its use on tumor resection planning and forecasting of eloquent involvement, since the good correlation of nTMS and direct cortical stimulation (Picht et al., 2016; Haddad et al., 2021; Sollmann et al., 2021). While the creation of cortical maps for surgical purposes is a consolidated matter, the use of objective parameters for quantifying the brain plasticity still needs further attention.

The aim of this study was to investigate possible measures that allows the evaluation of the magnitude of motor cortical plasticity induced by the presence of an intrinsic brain neoplasm in the vicinity of the motor cortex using nTMS.

## Materials and methods

### Setting

This prospective study recruited 35 adult patients with a single brain tumor with radiologic diagnosis of adult-type diffuse glioma in the vicinity of the pre-central gyrus, who underwent nTMS preoperative evaluations at a tertiary hospital in São Paulo, Brazil.

### Preoperative clinical evaluation and brain tumor management

Muscle strength was assessed preoperatively, and the motor score was defined as upper extremities strengths of each hemibody according to Medical Research Council grade scale. Performance status was evaluated using Karnofsky Performance Status and Eastern Cooperative Oncology Group Scales (ECOG). The tumors were graded in low-grade glioma (LGG) and high-grade glioma (HGG) according to the presence of necrosis and contrast enhancement on the MRI.

All patients underwent standard surgical treatment and final histopathological data was recorded and stratified by OMS grading and LGG and HGG.

### nTMS evaluation

During the week prior to the surgery participants underwent nTMS using structural MRI and infrared-based navigation system (Brainsight TMS 2.4.6, Canada) for guidance and registration of the stimulated points. The simulation was performed by applying single and paired pulses on the primary motor area (M1) corresponding to the cortical representation of hands and a radius of 5 cm around it, both on healthy and ill cerebral hemispheres, using a figure-of-eight stimulation coil (Magventure Tonica Elektronik, Farum, Denmark). Target points were identified when the hand movement was present using 65% TMS intensity. Muscle output due to cortical stimulation was evaluated using surface integrated electromyogram (EMG) electrodes attached to the muscles of hands (first dorsal interosseous) for determination of Resting Motor Threshold (RMT) as described previously in the literature (Rothwell et al., 1999; Groppa et al., 2012; Rossini et al., 2015). For the mapping registration of the response, considering the exploration of non M1 areas, single pulses were applied at 120% intensity of RMT and motor evoked potentials (MEP) were considered positive when any muscle twitch was present (all/none response). Then it was recorded if more than three activations out of five stimuli responses on the same point on a similar fashion of that performed during awake surgery (Morshed et al., 2021). All evaluations were performed by the same examiner.

### Data evaluation and graphic representation

Four parameters were determined for the evaluation of the plastic effects. All graphic and the extraction of the coordinates were executed using the Brainsight software. Firstly, a reference point was set on the posterior margin of the precentral gyrus, aligned with the “omega” representing M1 on both the affected and healthy hemispheres, it was set manually by the average visual marking of principal examiner and the referred surgeon of the case in order to compensate for tumoral distortion, the setting of the point was defined as the apex of the precentral “omega” on the border of the central sulcus, when the distortion did not allow for visual identification of the precentral gyrus margin, a standardized stereotactic MNI map was used. Then, the Euclidian center of gravity was calculated for the positive motor points on both hemispheres, creating a center of gravity (CG) point. The first parameter, ΔL was the distance between the reference point and CG. The second parameter was the dispersion (SDpc) from all positive points regarding CG.


$$S\,D\,p\,c=\sqrt{\frac{\begin{array}{c} (\sum_{a=1}^{n}(\sqrt{\left({\left(△\,P\,C\,{\text{X}}_{n}\right)}^{2}+{\left(△\,P\,C\,Y_{n}\right)}^{2}+{\left(△\,P\,C\,Z_{n}\right)}^{2}\right)}\\ -\frac{\sqrt{\left({\left(△\,P\,C\,{\text{X}}_{n}\right)}^{2}+{\left(△\,P\,C\,Y_{n}\right)}^{2}+{\left(△\,P\,C\,Z_{n}\right)}^{2}\right)}}{n})){}^{2} \end{array}}{n}}$$

- SDpc: Dispersion (standard deviation) of all positive stimulated points.

- ΔPC: distance from positive stimulated point to center of gravity in each orthogonal plane.

- n: total of positive stimulated points.

- X_*n*_; Y_*n*_; Z_*n*_: each orthogonal x,y,z axis reference for each positive stimulated point.

The third parameter was the variability of the same points (VCpc).


$$V\,C\,p\,c=\frac{S\,D\,p\,c*100}{\frac{\sqrt{\left({\left(△\,P\,C\,{\text{X}}_{n}\right)}^{2}+{\left(△\,P\,C\,Y_{n}\right)}^{2}+{\left(△\,P\,C\,Z_{n}\right)}^{2}\right)}}{n}}$$

- VCpc: Variability (Variation Coefficient) of distances from each positive stimulated point to the center of gravity.

- SDpc: Dispersion (standard deviation) of all positive stimulated points.

- ΔPC: distance from stimulated point to center of gravity in each orthogonal plane.

- n: total of positive stimulated points.

- X_*n*_; Y_*n*_; Z_*n*_: each orthogonal x,y,z axis reference for each positive stimulated point.

**FIGURE 1:**
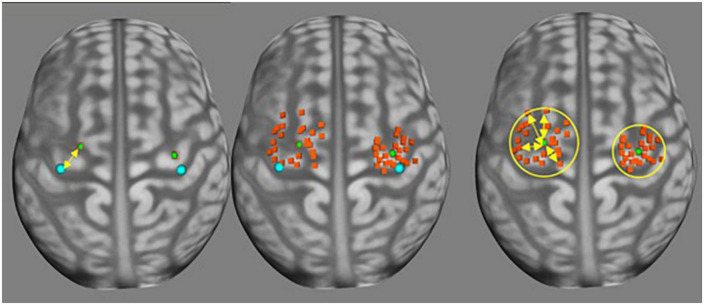
Demonstration of ΔL, SDpc, and VCpc over a standard tridimensional brain reconstruction. The blue dots are the reference points, green dots are CG points. The yellow arrow in the first panel represents ΔL. Orange points in the second and third panels are positive motor points. In the third panel, the yellow circle depicts an approximation of SDpc and the yellow arrows are the distances used for obtaining VCpc.

Finally, the RMT was defined as the fourth parameter. CG, ΔL, SDpc, and VCpc are graphically represented on Figure 1.

All values were compared by the ratio between the ill and healthy hemispheres. In order to access the magnitude of the difference, all ratios were fraction adjusted to values greater than 1.0. This mathematical maneuver allowed the comparison of the “amount” of change, regardless of the qualitative direction or characteristics of plasticity, i.e.: a ratio of 1.2 represents a difference of 20% among the ill and healthy hemispheres but does not imply it was an increase or decrease on the affected hemisphere. For the qualitative analysis, a graphic representation was used, and categorical factors regarding the affected hemisphere were defined. Closing and Distancing for ΔL; Dispersion and Concentration for SDpc; Homogeneous and Heterogeneous for VCpc; Increased or Decreased for RMT. Additional direction information–anterior, posterior, medial, lateral–was obtained by this analysis.

Due to the absence of data on most of the studied ratio parameters in healthy population, the reference for the expected normal ratios was obtained by the calculation of the normalized ratio of the average of all left healthy over the average of all right healthy hemispheres studied.

### Ethical standard

This study was approved by Ethics and Research Committee of University of Sao Paulo Medical School and all individuals have signed an informed consent.

### Statistical analysis

We compared the patients’ ratios with the control set using the Mann–Whitney U test. The Kruskal–Wallis test was performed to compare the studied variables regarding multiple categorical characteristics such as motor grading and performance status. Comparisons among the subgroups of patients according to brain tumor histopathology diagnosis [LGG vs. HGG and World Health Organization (WHO) grade I, II and III gliomas vs. glioblastomas (GBMs)] were performed using the Mann–Whitney test. Continuous variable normality was verified using Kolmogorov–Smirnov test. The analyses were performed using the Statistical Package for Social Sciences, version 28.0 (IBM Statistics, Armonk, NY, USA). The data were considered significant when *p* was < 0.05.

## Results

Thirty-five patients were evaluated with complete nTMS and histopathological results. General characteristics of the patients are presented in Table 1. There were 23 male and 12 female patients with a mean age of 48.2 ± 15.9. The median KPS was 90 (ranging from 60 to 100) and the ECOG was 0 (range 3-0). The presence of motor deficit was observed in 15 patients (42.9%) with strengths 3 and 4, and none of the patients were complete or functionally hemiplegic. As both initial and ongoing symptom 17 patients presented seizures (48.6%), while 77% were in use of some antiepileptic drug. The preoperative assessment of the MRI indicated 23 patients with low-grade gliomas, while the histopathological results demonstrated 15 low-grade gliomas, all the eight patients with a final higher grade than expected presented a grade III tumor. Astrocytoma was the most common tumor type (15 patients, 42.9%), followed by glioblastoma (11 patients, 31.4%). All patients were naïve regarding not only brain tumor treatment but also any brain injuries, surgeries or intracranial treatment.

**TABLE 1:** General characteristics of the study sample.

Seq.	Age	Gender	Radiologic grading	Histopathologic grading	WHO grad.	Histopath. grad.	IDH 1	ATRX	1p19q	Side	KPS	ECOG	Hemip.	Strength degree	Seizures
1	47	F	LGG	Oligodendroglioma	II	LGG	Mutated	Preserved	Deleted	Right	100	0	Yes	4	Yes
2	45	M	LGG	Oligodendroglioma	II	LGG	Mutated	Preserved	Deleted	Right	90	1	No	5	No
3	33	M	LGG	Astrocitoma	III	HGG	Wild type	Absent	N/A	Left	100	0	No	5	No
4	21	M	LGG	Astrocitoma	III	HGG	Wild type	Absent	N/A	Left	90	1	No	5	Yes
5	46	M	LGG	Astrocitoma	II	LGG	Mutated	Preserved	N/A	Right	90	1	No	5	No
6	79	F	HGG	Glioblastoma	IV	HGG	Wild type	N/A	N/A	Left	80	1	Yes	4	No
7	47	F	LGG	Astrocitoma	II	LGG	Mutated	Preserved	N/A	Right	100	0	No	5	Yes
8	37	M	LGG	Astrocitoma	II	LGG	Mutated	Absent	N/A	Right	100	0	No	5	No
9	18	M	LGG	Ganglioglioma	I	LGG	N/A	N/A	N/A	Right	100	0	No	5	No
10	60	F	LGG	Astrocitoma	III	HGG	Wild type	Absent	N/A	Left	90	0	No	5	Yes
11	46	F	LGG	Astrocitoma	II	LGG	Mutated	Absent	N/A	Left	90	0	No	5	No
12	57	F	LGG	Astrocitoma	II	LGG	Mutated	Absent	N/A	Right	100	0	No	5	Yes
13	59	F	LGG	Oligodendroglioma	III	HGG	Mutated	Preserved	Deleted	Right	70	1	No	5	Yes
14	46	M	LGG	Oligodendroglioma	III	HGG	Mutated	Preserved	Deleted	Right	80	1	Yes	4	Yes
15	38	M	LGG	Astrocitoma	II	LGG	Mutated	Absent	N/A	Right	90	0	No	5	No
16	53	F	LGG	Astrocitoma	III	HGG	Wild type	Absent	N/A	Left	90	1	Yes	4	No
17	37	M	LGG	Oligodendroglioma	II	LGG	Mutated	Preserved	Deleted	Left	90	0	No	5	Yes
18	31	M	LGG	Astrocitoma	II	LGG	Mutated	Preserved	N/A	Left	100	0	No	5	Yes
19	31	M	LGG	Astrocitoma	II	LGG	Mutated	Absent	N/A	Right	100	0	No	5	Yes
20	35	M	LGG	Astrocitoma	II	LGG	Mutated	Absent	N/A	Right	100	0	No	5	Yes
21	24	M	LGG	Astrocitoma	III	HGG	Wild type	Absent	N/A	Right	100	0	Yes	4	No
22	61	F	HGG	Glioblastoma	IV	HGG	Wild type	N/A	N/A	Right	60	3	Yes	3	No
23	51	M	HGG	Glioblastoma	IV	HGG	Wild type	Preserved	N/A	Left	70	2	Yes	3	Yes
24	42	M	HGG	Glioblastoma	IV	HGG	Wild type	Preserved	N/A	Left	90	0	No	5	No
25	69	M	HGG	Glioblastoma	IV	HGG	Wild type	N/A	N/A	Right	90	2	Yes	3	No
26	51	M	HGG	Glioblastoma	IV	HGG	Wild type	Preserved	N/A	Left	80	1	Yes	4	Yes
27	72	M	HGG	Glioblastoma	IV	HGG	Wild type	Preserved	N/A	Right	80	2	Yes	3	No
28	63	M	HGG	Glioblastoma	IV	HGG	Wild type	N/A	N/A	Right	90	1	Yes	4	Yes
29	74	M	HGG	Glioblastoma	IV	HGG	Wild type	N/A	N/A	Left	80	1	Yes	4	No
30	79	F	HGG	Glioblastoma	IV	HGG	Wild type	N/A	N/A	Right	70	3	Yes	3	No
31	60	F	HGG	Glioblastoma	IV	HGG	Wild type	N/A	N/A	Right	80	2	Yes	4	No
32	48	M	LGG	Oligodendroglioma	II	LGG	Mutated	Preserved	Deleted	Left	90	0	No	5	No
33	52	M	HGG	Oligodendroglioma	III	HGG	Mutated	Preserved	Deleted	Right	90	0	No	5	Yes
34	27	F	LGG	Astrocitoma	II	LGG	Mutated	Preserved	N/A	Right	100	0	No	5	Yes
35	47	M	LGG	Oligodendroglioma	III	HGG	Mutated	Preserved	Deleted	Left	90	1	Yes	4	Yes

We observed that the adjusted ratios of ΔL, SDpc, VCpc, and RMT were significantly different from the expected normality. Suggesting a good capacity to identify neuroplastic changes, detailed in Table 2.

**TABLE 2:** Evaluation of significance and distribution of rΔL, rSDpc, rVCpc, and rRMT related to the control set.

Variable	Reference healthy ratio	Median	Q1	Q3	*P*
rΔL	1.0006	1.363	1.193	1.995	<0.001
rSDpc	1.0062	1.1170	1.041	1.301	<0.001
rVCpc	1.0041	1.214	1.134	1.318	<0.001
rRMT	1.0000	1.143	1.075	1.250	<0.001

rΔL, ratio of distances from reference point to center of gravity; rSDpc, ratio of dispersion; rVCpc, ratio of variability; rRMT, ratio of resting motor threshold.

**FIGURE 2:**
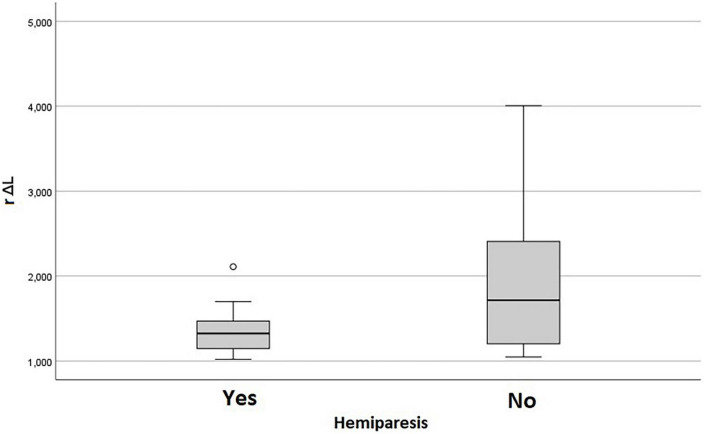
Box plot graph of rΔL and hemiparesis.

**FIGURE 3:**
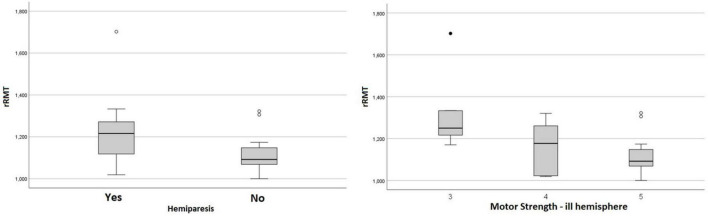
Box plot graph of rRMT and hemiparesis and motor scores.

**FIGURE 4:**
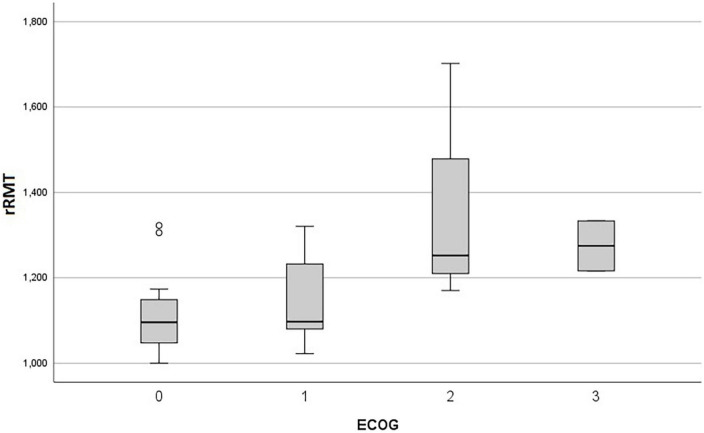
Box plot graph of rRMT and ECOG.

Lower values of rΔL and higher values of rRMT were related to the presence of hemiparesis (*p* = 0.036 and *p* = 0.028, respectively), and the rRMT was also related to the grading of motor strength (*p* = 0.019) as shown in the Figures 2, 3 The performance status, and more importantly the ECOG value related directly to the rRMT (*p* = 0.028, Figure 4). There were no significant difference on rRMT regarding the occurrence of seizures, use of antiepileptic drugs.

**FIGURE 5:**
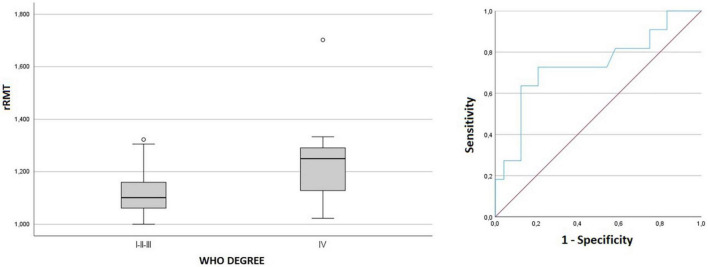
Box plot and ROC curve of rRMT and tumor grading.

**FIGURE 6:**
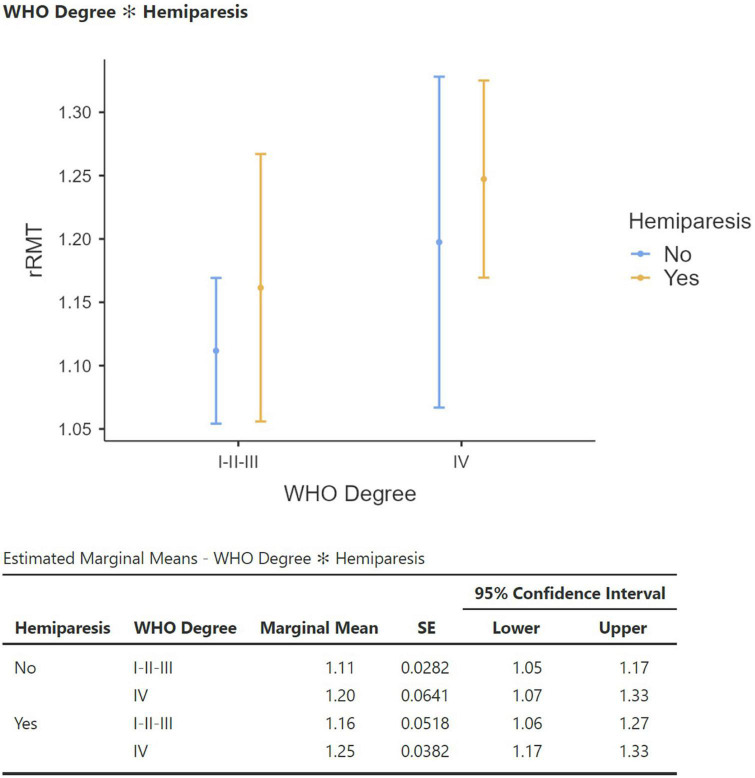
Estimated marginal means graph of linear regression of rRMT, tumor grading, and hemiparesis.

Comparing the tumor grading, no statistically significant difference was found between LGG and HGG regarding any of the ratios parameters. We observed rRMT values were significantly higher in grade IV tumors compared to grades I, II and III (*p* = 0.025, Figure 5). A ROC analysis estimation determined an AUC of 0.737 with a threshold value of 1.169 rRMT value as cutoff for grade IV HGG. The analysis of independent factors to evaluate hemiparesis demonstrated a milder tendency of higher rRMT values in high grade gliomas, but with no statistical significance (grade *p* = 0.167/hemiparesis *p* = 0.388, Figure 6).

**FIGURE 7:**
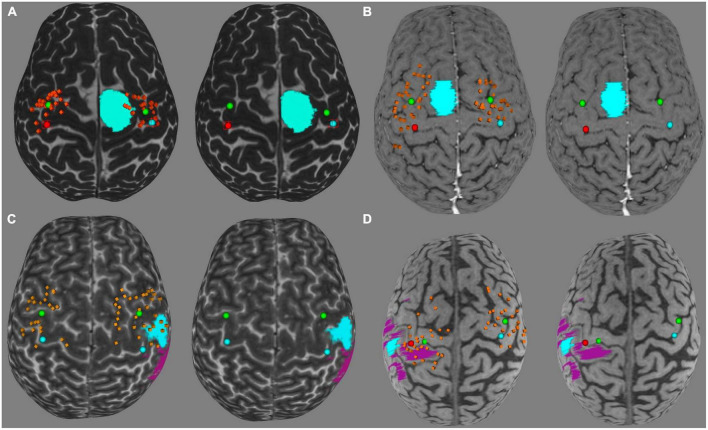
Graphic representation of four patients **(A–D)** mapping. The red and blue dots are the reference points, green dots are CG points, orange points are positive motor points. Light blue areas are cortical tumor delimitation and purple areas represents edema. Example of qualitative analysis on the tumor hemisphere compared to the healthy hemisphere: **(A)** closing, Homogeneous; **(B)** lateral, dispersion, Heterogeneous; **(C)** distancing, Heterogeneous; **(D)** posterior, medial, dispersion.

There was no relation observed to ratios of ΔL, SDpc, VCpc, and RMT to the location of the tumor, when studied both absolute–frontal lobe and parietal lobe–and relative–anterior, posterior, lateral or medial to the reference point. Also there was no pattern regarding the location or grading of the tumor and the qualitative plastic changes. On an individual case level, the graphic representation allowed the surgeon to access the direction of the motor points displacement, as demonstrated with representative cases in Figure 7.

## Discussion

This study sums to the current knowledge of brain plasticity in the context of brain tumors involving the primary motor cortex by evaluating graphic cortical maps representations already reported, as well as describing adjusted quantitative parameters as rRMT and, introducing ratio values of ΔL, SDpc, VCpc as possible markers of brain plasticity. The use of the normalized ratios was to our knowledge a novelty, and despite masking the qualitative evaluation of the absolute parameters, allows for a better interpatient comparison, mitigating previous dissonances in literature, such as reports of both higher and lower RMT ratios in the similar groups of glioma patients (Lavrador et al., 2020; Gomes dos Santos et al., 2021).

Regarding the dislocation of the CG, Conway et al. (2017) described that tumors located anterior to the precentral gyrus tend to generate displacements toward the tumor, while other authors saw the same effect but for tumors located in the parietal lobe (Takahashi et al., 2013; Bulubas et al., 2018), in both cases, the amount of displacement was not directly associated with the motor status. Our study focused on sheer difference among both sides, demonstrating that smaller rΔL were associated the presence of hemiparesis, the mechanism underlying these observations are still unclear, but two main hypothesis were be addressed during the evaluation of these patients: the first one is that despite the direction of the CG displacement, higher rΔL might represent slower and more important changes in the cortical representation, making the preservation of the motor strength possible, similarly to what is observed during long term rehabilitation (Desmurget et al., 2007). The second hypothesis considers that pathways of white matter tracts might also be affected and the migration of these pathways might recruit more sparse and farther cortical neurons (Duffau, 2022).

The capabilities of nTMS mapping regarding brain glioma surgery and it’s impacts on prognosis have been thoroughly demonstrated during the last years (Picht et al., 2016; Haddad et al., 2021; Schiavao et al., 2022). Our observation that the rRMT relates to the performance status reinforces that the preoperative mapping might someday be integrated to a broader prognostic tool. The grade IV tumor was also associated with higher rRMT, and while the possibility of tumor grade prediction by nTMS has been previously described (Lavrador et al., 2020; Neville et al., 2021), we propose an initial take on cutoff values that might be useful as an aid to the imaging methods, specifically for differentiating WHO grade IV tumors. The occurrence of hemiparesis alone was an important confounding factor, but even though there was still a tendency for higher rRMT values for grade IV tumors. Additionally, even though the remaining parameters were not significantly different among tumor grades, further studies with larger samples might also provide information for the clinically impactful differentiation of grade II and III tumors.

A limitation of our study is that the MEP and the motor twitch were only considered for hands and upper limbs, which might hinder the full evaluation of the motor system related gliomas. Another aspect that may be considered a limitation to our method was the use of the motor twitch alone for the creation of the map, while it allows for a broader evaluation of non-primary areas and was compensated by intrinsic control with the healthy hemisphere, a markedly high MEP might dislocate the CG in different patterns, the use of a MEP amplitude pondered CG might prove useful in future studies. Finally, despite the consecutive selection of patients within our service the non-evaluation of patients who were not candidates for surgery and the absence of patients with more severe motor deficits reflect a possible selection bias. The nature of a single evaluation precludes us from the evaluation of possible correlations of the plasticity parameters studied and the outcome of the treatment.

## Conclusion

The nTMS was able to quantitatively and qualitatively demonstrate the occurrence of brain plasticity induced by an intrinsic brain tumor. The graphic evaluation allowed the observation of useful characteristics for the operative planning, while the mathematical analysis made it possible to quantify the magnitude of the plasticity.

## Data availability statement

The raw data supporting the conclusions of this article will be made available by the authors, without undue reservation.

## Ethics statement

The studies involving human participants were reviewed and approved by the Ethics and Research Committee of the University of São Paulo Medical School. The patients/participants provided their written informed consent to participate in this study. Written informed consent was obtained from the individual(s) for the publication of any potentially identifiable images or data included in this article.

## Author contributions

CA, IN, CH, and WP: conception and design. IN and WP: patients recruitment. CA, CH, and AG: data collection. CA, IN, and WP: data analysis and interpretation. CA and IN: drafting the manuscript for important intellectual content. CH, AG, MT, and AB: text review. CA, IN, CH, AG, AB, MT, and WP: final approval. All authors contributed to the article and approved the submitted version.
